# Functional characterisation of the TSC1–TSC2 complex to assess multiple *TSC2 *variants identified in single families affected by tuberous sclerosis complex

**DOI:** 10.1186/1471-2350-9-10

**Published:** 2008-02-26

**Authors:** Mark Nellist, Őzgür Sancak, Miriam Goedbloed, Alwin Adriaans, Marja Wessels, Anneke Maat-Kievit, Marieke Baars, Charlotte Dommering, Ans van den Ouweland, Dicky Halley

**Affiliations:** 1 Department of Clinical Genetics, Erasmus Medical Centre, Rotterdam, The Netherlands; 2 Department of Clinical and Human Genetics, Free University Medical Centre, Amsterdam, The Netherlands

## Abstract

**Background:**

Tuberous sclerosis complex (TSC) is an autosomal dominant disorder characterised by seizures, mental retardation and the development of hamartomas in a variety of organs and tissues. The disease is caused by mutations in either the *TSC1 *gene on chromosome 9q34, or the *TSC2 *gene on chromosome 16p13.3. The *TSC1 *and *TSC2 *gene products, TSC1 and TSC2, interact to form a protein complex that inhibits signal transduction to the downstream effectors of the mammalian target of rapamycin (mTOR).

**Methods:**

We have used a combination of different assays to characterise the effects of a number of pathogenic TSC2 amino acid substitutions on TSC1–TSC2 complex formation and mTOR signalling.

**Results:**

We used these assays to compare the effects of 9 different TSC2 variants (S132C, F143L, A196T, C244R, Y598H, I820del, T993M, L1511H and R1772C) identified in individuals with symptoms of TSC from 4 different families. In each case we were able to identify the pathogenic mutation.

**Conclusion:**

Functional characterisation of TSC2 variants can help identify pathogenic changes in individuals with TSC, and assist in the diagnosis and genetic counselling of the index cases and/or other family members.

## Background

Tuberous sclerosis complex (TSC) is an autosomal dominant disorder characterised by seizures, mental retardation and the development of hamartomas in a variety of organs and tissues [1]. The disease is caused by mutations in either the *TSC1 *gene on chromosome 9q34 [2], or the *TSC2 *gene on chromosome 16p13.3 [3]. Loss of heterozygosity studies at the *TSC1 *and *TSC2 *loci in TSC-associated lesions indicate that *TSC1 *and *TSC2 *are tumour suppressor genes [1]. Comprehensive screens for mutations at both the *TSC1 *and *TSC2 *loci have been performed in several large cohorts of TSC patients and a wide variety of different pathogenic mutations have been described [4-10]. In most studies approximately 20% of the identified mutations are either missense changes or small, non-truncating insertions/deletions, predominantly in the *TSC2 *gene.

The phenotypic expression of TSC is highly variable and in some cases it can be difficult to establish a definitive clinical diagnosis. Generally the diagnosis is made based on multiple clinical criteria that are categorized into major and minor features [11]. The presence of 2 major features, or one major and 2 minor features, is sufficient for a definite diagnosis. In recent years, mutation analysis has become an additional diagnostic tool in familial as well as sporadic TSC. However, it is sometimes difficult to establish whether an identified nucleotide change, particularly a missense change, is a genuine pathogenic mutation, or a (rare) polymorphism. In familial cases where a missense change cosegregates with TSC, or in cases where key relatives are not available for testing, a distinction cannot be made on genetic grounds alone. Furthermore, many tools for the analysis of amino acid substitutions [12-14], may not predict the effect of a particular substitution reliably.

The *TSC1 *and *TSC2 *gene products, TSC1 and TSC2, interact to form a protein complex which acts as a GTPase activating protein (GAP) for the rheb GTPase, preventing the rheb-GTP-dependent stimulation of cell growth through the mammalian target of rapamycin (mTOR) [15]. In cells lacking either *TSC1 *or *TSC2*, the downstream targets of mTOR, p70 S6 kinase (S6K) and ribosomal protein S6, are constitutively phosphorylated [16,17]. The identification of the role of the TSC1–TSC2 complex in regulating signal transduction through mTOR has made it possible to assess the activity of different TSC1 and TSC2 variants. The effects of amino acid changes on TSC1–TSC2 complex formation, on the activation of rheb GTPase activity by the complex, and on the phosphorylation status of S6K and S6, the downstream effectors of mTOR, can be determined [18,19].

Here, we apply assays of TSC1–TSC2 function to assist in the identification, diagnosis and counselling of 4 families with TSC. In each index case at least 2 changes in the *TSC2 *gene were detected. To identify the disease-causing mutation in each family we characterised the effects of the changes on the activity of the TSC1–TSC2 complex. In each case we were able to identify the pathogenic *TSC2 *variant. Our analysis demonstrates that biochemical assays can help resolve otherwise intractable problems in clinical genetic diagnostics.

## Methods

### Mutation analysis

DNA was extracted from peripheral blood using standard techniques. Mutation analysis was performed using a combination of single-strand conformational polymorphism analysis, denaturing gradient gel electrophoresis, direct sequencing, Southern blotting and fluorescence *in situ *hybridisation, as previously described [8]. In addition, the multiplex ligation-dependent probe amplification assay was performed (MRC Holland, The Netherlands). *TSC2 *sequence changes were numbered according to the original cloning paper [3].

To investigate whether the changes had an effect on RNA splicing, 3 different splice-site prediction programs [12-14]. were used. Amino acid substitutions were evaluated using the PAM 250 [15], BLOSUM 62 [16] and Grantham [17] matrices and SCANSITE [23].

### Generation of constructs and antisera

Expression constructs encoding the identified variants were derived using the Stratagene QuikChange site-directed mutagenesis kit and in each case verified by sequencing the complete open reading frame. All the other constructs used in this study have been described previously [21,24,25]. Polyclonal rabbit antisera specific for human TSC1 and TSC2 have been described previously [25]. Other antibodies were purchased from Cell Signaling Technology.

Transfections, immunocytochemistry, immunoblotting, immunoprecipitation assays and the *in vitro *assay of rheb GTPase activity were performed as described previously [21].

## Results

### Patient characteristics

#### Family 1

**Figure 1:**
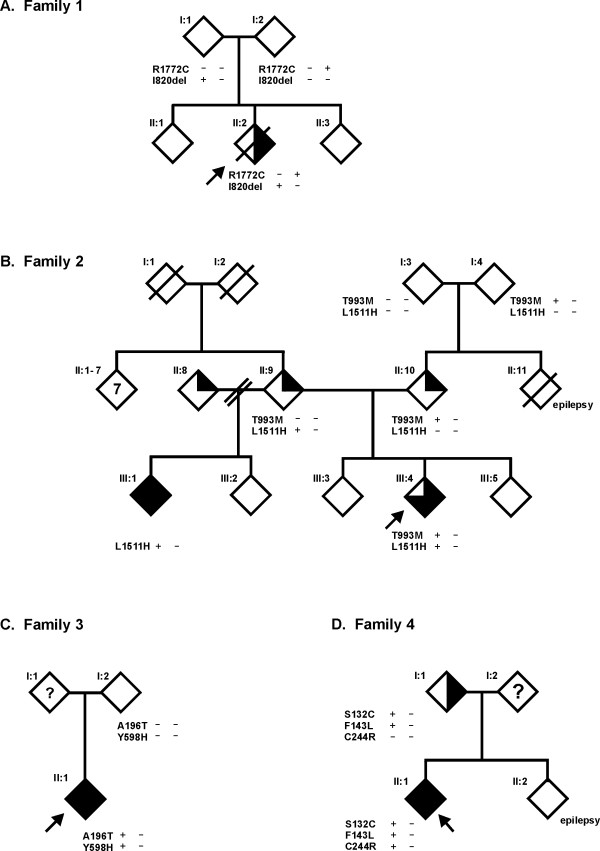
Pedigrees of the investigated families. (A) Family 1, (B) Family 2, (C) Family 3 and (D) Family 4. Arrows indicate the index cases. Clear symbols indicate no signs or symptoms of TSC; 1/4-filled symbols indicate one minor feature of TSC; 1/2-filled symbols indicate possible TSC; 3/4-filled symbols indicate probable TSC, filled symbols indicate definite TSC, and individuals with epilepsy only are indicated. A question mark indicates individuals where no clinical data was available. Genotypes are shown for the individuals where DNA was available for testing.

(Figure 1A) The index case (II:2) died shortly after birth due to a solitary rhabdomyoma in the right ventricle of the heart. Post-mortem examination did not reveal any other findings indicative of TSC. Both parents underwent a full clinical evaluation. Dermatological, cardiological, ophthalmological, neurological and radiological examinations were negative for signs of TSC except for one nail groove and one hypomelanotic macule in individual I:1. The sibling of the index case (II:1) was healthy, but was not investigated further. At the first trimester of the third pregnancy, mutation analysis of the index case had not been completed and prenatal DNA testing could not be offered. Fetal echocardiography did not reveal any heart defects and the pregnancy resulted in the birth of a healthy child (II:3).

#### Family 2

(Figure 1B) The index case (III:4) had multiple hypomelanotic macules and dental pits, epilepsy and electroencephalographic abnormalities, and was diagnosed with 'probable TSC'. One of the parents of the index case (II:10) had multiple dental pits and a computer tomography scan revealed 2 small calcifications in the nucleus caudatus which were not typical for TSC. Magnetic resonance imaging was performed and showed no additional abnormalities. Individual II:9, the other parent of the index case, had a single 2 centimetre cyst in the left kidney and a single ash leaf-shaped area of hypopigmentation, insufficient for a diagnosis of TSC. Individual II:11, the sibling of II:10, had neurological and ophthalmological problems and died at the age of 13 due to status epilepticus. Individual III:1, the half-sibling of the index case and the child of individual II:9, had a history of possible epilepsy at the age of 3 years and was again diagnosed with epilepsy at 12 years of age. A subsequent full diagnostic work-up identified cortical tubers and 7 hypomelanotic macules, fulfilling the diagnostic criteria for definite TSC. Individual II:8 had multiple dental pits and 10 irregular hypopigmentations, atypical for TSC.

#### Family 3

(Figure 1C) The index case (II:1) was diagnosed with definite TSC. One parent (I:2) did not show any clinical signs of TSC, and there was no information on the other parent (I:1).

#### Family 4

(Figure 1D) The index case (II:1) was diagnosed with definite TSC. Individual I:1, the parent of the index case, had angiomyolipoma but no other reported signs of TSC, and individual II:2, the sibling of the index case had epilepsy, but no other signs of TSC.

### Mutation analysis

#### Family 1

The index case (II:2) was heterozygous for 2 changes in the *TSC2 *gene: *TSC2 *2476delATC (deletion of isoleucine at codon 820, I820del) and *TSC2 *5332C>T (arginine to cysteine substitution at codon 1772, R1772C). Analysis of the parents indicated that the index case had inherited one change from each parent. No DNA was available from other relatives.

#### Family 2

The index case (III:4) was heterozygous for 2 missense changes: *TSC2 *2996C>T (threonine to methionine substitution at codon 993, T993M) and *TSC2 *4550T>A (leucine to histidine amino acid substitution at position 1511, L1511H). Analysis of additional family members (individuals I:3, I:4, II:9, II:10 and III:3) confirmed that one substitution was paternal in origin and the other maternal.

#### Family 3

The index case (II:1) was heterozygous for 2 missense changes: *TSC2 *604G>A (alanine to threonine substitution at codon 196, A196T) and *TSC2 *1810T>C (tyrosine to histidine substitution at codon 598, Y598H). Neither substitution was detected in parent I:2, and DNA from parent I:1 was not available for testing.

#### Family 4

The index case (II:1) was heterozygous for 3 missense changes in the *TSC2 *gene: *TSC2 *413C>G (serine to cysteine substitution at codon 132, S132C), *TSC2 *447C>G (phenylalanine to leucine substitution at codon 143, F143L) and *TSC2 *748T>C (cysteine to arginine substitution at codon 244, C244R). The S132C and F143L substitutions were inherited from parent I:1. No DNA was available from the other parent.

Comparison of the allele ratios of the index cases and parents did not reveal any evidence for somatic mosaicism in the leukocyte DNA from any of the families studied. None of the changes showed an effect on splicing according to the 3 splice-site prediction programs used.

As shown in Table 1, the identified amino acid changes were compared using the PAM 250 [15], BLOSUM 62 [16] and Grantham matrices [17]. In addition, the degree of conservation of the amino acid residues in different metazoan species was compared, as shown in Table 2.

**Table 1:** Scores of the BLOSUM 62, PAM 250 and Grantham matrices. BLOSUM 62 scores range between -4 and 11, PAM 250 scores between -8 and 17, and Grantham scores between 5 and 215. For the BLOSUM 62 and PAM 250 matrices, a more negative score corresponds to a less conservative amino acid change. For the Grantham matrix, a higher number reflects a less conservative change.

**Amino Acid Substitution**	**Family**	**BLOSUM 62**	**PAM 250**	**Grantham**
TSC2 R1772C	1	-3	-4	180
TSC2 T993M	2	-1	-1	81
TSC2 L1511H	2	-3	-2	99
TSC2 A196T	3	0	1	58
TSC2 Y598H	3	2	0	83
TSC2 S132C	4	-1	0	112
TSC2 F143L	4	0	2	22
TSC2 C244R	4	-3	-4	180

**Table 2:** Evolutionary conservation of the variant TSC2 amino acids. Inter-species conservation of the TSC2 S132, F143, A196, C244, Y598 I820, T993, L1511 and R1772 amino acids is shown.

**Family 1**			
	I820	R1772	
human	PDII**I**KALP	GQRK**R**LISS	
mouse	PDII**I**KALP	GQRK**R**LISS	
rat	PDII**I**KALP	GQRK**R**LISS	
pufferfish	PDIM**I**KLLP	GQRK**R**LVST	
fruitfly	PEALMRKLP	no homology	
			
**Family 2**			
	T993	L1511	
human	SRIQ**T**SLTS	SVQL**L**DQIP	
mouse	SRIQ**T**SLTS	SVQL**L**DQIP	
rat	SRIQ**T**SLTS	SVQL**L**DQIP	
pufferfish	RRMH**T**STTT	AVKV**L**DQMP	
fruitfly	no homology	AVSLIDLVP	
			
**Family 3**			
	A196	Y598	
human	DEYI**A**RM V	IQLH**Y**KHSY	
mouse	DEYI**A**SM V	IQLH**Y**KHGY	
rat	DEYI**A**PM V	IQLH**Y**KHGY	
pufferfish	DQNV**A**SM V	LQLH**Y**KNKY	
fruitfly	DKDILVGIV	LELH**Y**ERPK	
			
**Family 4**			
	S132	F143	C244
human	KDYP**S **NED	RLEV**F**KALT	IVTL**C**RTIN
mouse	KDYP**S **NED	RLEV**F**KALT	IITL**C**RTIN
rat	KDYP**S **NED	RLEV**F**KALT	IITL**C**RTVN
pufferfish	RDYQPCNED	RLEV**F**KALT	IITL**C**RTVN
fruitfly	IQNHEARED	LLELLDTLT	ITTL**C**RTVN

### Functional analysis

#### Families 1 and 2

The genetic data from families 1 and 2 indicated that in both families the index case had inherited a different, rare *TSC2 *variant from each parent. To determine whether the I820del, R1772C, T993M and L1511H variants corresponded to pathogenic mutations, the biochemical activity of each variant was compared to wild-type TSC2 and a known, pathogenic TSC2 missense variant (R611Q) using a variety of functional assays.

**Figure 2:**
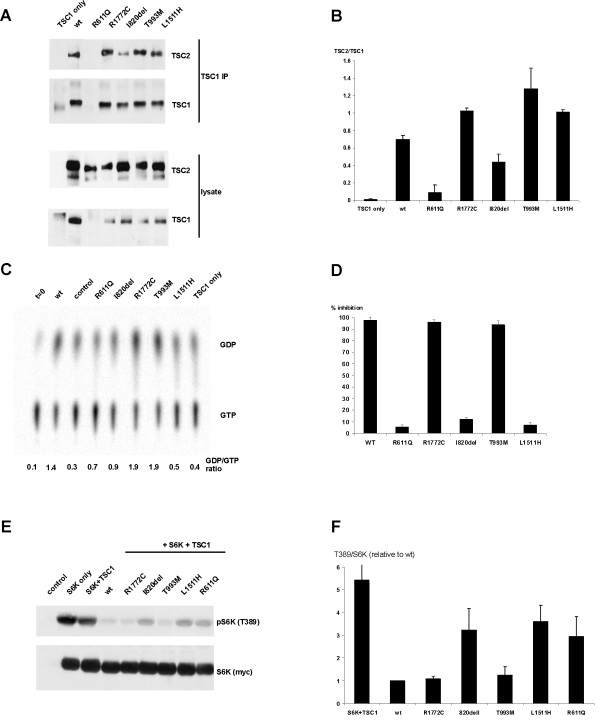
Results of the functional assays on the TSC2 variants identified in family 1 (variants I820del and R1772C) and family 2 (variants T993M and L1511H). (A) Interaction between TSC1 and TSC2 variants. TSC1–TSC2 complexes were immunoprecipitated with antibodies specific for exogenous TSC1 from HEK 293 cells over-expressing TSC1 and wild-type TSC2 (wt) or TSC1 and the TSC2 variants. (B) Interaction between TSC1 and TSC2 variants. Ratio of coimmunoprecipitated TSC2:TSC1, as detected by immunoblotting, was estimated by densitometry scanning (Total Scan). (C) *In vitro *rheb GAP activity of immunoprecipitated TSC1–TSC2 complexes. Rheb-bound GDP/GTP ratios were determined after 90 minutes incubation with the immunoprecipitated wild-type TSC1–TSC2 complex (wt), protein A beads only (control), TSC1 only, or TSC1–TSC2 variant complexes. Rheb-bound GDP/GTP prior to addition of the TSC1–TSC2 complexes is shown (t = 0). (D) Inhibition of S6 phosphorylation in Tsc2 -/- MEFs. Percentage of *Tsc2 *-/- MEFs transfected with expression constructs encoding TSC1 and wild-type TSC2, or TSC1 and the TSC2 variants, and showing inhibition of S6 phosphorylation. Data from at least 3 separate experiments are shown. (E) TSC2-dependent inhibition of S6K-T389 phosphorylation. S6K, TSC1 and wild-type TSC2 (wt), or S6K, TSC1 and the TSC2 variants, were coexpressed in HEK 293 cells. Phosphorylation of S6K at the T389 position was determined by immunoblotting. A representative example of at least 3 separate experiments is shown. (E) TSC2-dependent inhibition of S6K-T389 phosphorylation. Ratio of total S6K:T389-phosphorylated S6K, as detected by immunoblotting, was estimated by densitometry scanning (Total Scan). Mean ratios relative to TSC2 wild-type (wt) are shown.

To investigate the ability of the TSC2 I820del, R1772C, T993M and L1511H variants to interact with TSC1, coimmunoprecipitation experiments were performed using antibodies specific for TSC1. As shown in Figures 2A and 2B, coimmunoprecipitation of the TSC2 I820del variant was reduced compared to wild-type TSC2, but was not prevented completely (compare the I820del variant to the R611Q variant). The R1772C, T993M and L1511H amino acid substitutions did not reduce TSC1–TSC2 coimmunoprecipitation.

Next, the activation of rheb GTPase activity by the immunoprecipitated variant TSC1–TSC2 complexes was assayed. As shown in Figure 2C, in the presence of the wild-type TSC1–TSC2 complex the ratio of rheb-bound GDP to GTP was 3-fold higher than in the presence of TSC1 alone (control GDP/GTP = 0.4; wild-type GDP/GTP = 1.4). Coimmunoprecipitation of the TSC2 I820del variant was reduced compared to wild-type TSC2, and consequently the activation of rheb GTPase activity was also reduced (wild-type GDP/GTP = 1.4; I820del GDP/GTP = 0.9).

The immunoprecipitated R1772C and T993M variant complexes increased the GDP/GTP ratio more than 4-fold above the control value, and were therefore both at least as active as wild-type TSC2 (wild-type GDP/GTP = 1.4; R1772C and T993M GDP/GTP = 1.9). In contrast, although TSC1–TSC2 coimmunoprecipitation was unaffected by the L1511H substitution, the activation of rheb GTPase activity was reduced 3-fold compared to wild-type (wild-type GDP/GTP = 1.4; L1511H GDP/GTP = 0.5).

To determine whether the TSC2 I820del, R1772C, T993M and L1511H variants affected mTOR activity, the variants were overexpressed together with TSC1 and S6K in human embryonal kidney 293 cells. Phosphorylation of the exogenous S6K linker domain (T389), as detected by immunoblotting, was used as a read-out for mTOR activity. As shown in Figure 2E and 2F, S6K T389 phosphorylation was increased in the presence of the TSC2 I820del, L1511H and R611Q variants, compared to wild-type TSC2 and the R1772C and T993M variants. To confirm these findings, TSC1 and the TSC2 variants were overexpressed in *Tsc2 *-/- mouse embryo fibroblasts (MEFs) [16]. The S235/S236 phosphorylation of S6 in the MEFs expressing the TSC2 variants was determined by double-label immunofluorescent microscopy, as described previously [21]. As shown in Figure 2D, expression of the TSC2 R1772C and T993M variants suppressed S6 phosphorylation in more than 90% of the transfected cells, similar to wild-type TSC2. In contrast, less than 20% of the MEFs expressing the TSC2 I820del, L1511H or R611Q variants showed inhibition of S6 phosphorylation. Therefore, in both assays, the TSC2 I820del and L1511H variants were unable to inhibit mTOR, indicating that both these variants are pathogenic, while the TSC2 R1772C and T993M variants were just as active as wild-type TSC2 and are therefore not pathogenic amino acid substitutions.

#### Families 3 and 4

In families 3 and 4, the clinical and genetic data was incomplete. Multiple *TSC2 *variants were identified in the index cases, but it was not clear if they were *de novo*, or had been inherited from an untested parent. It was also unclear whether the changes were confined to the same allele. Therefore, to investigate the effects of the changes on the ability of the TSC1–TSC2 complex to antagonise mTOR signalling, TSC2 variants containing the different combinations of amino acid substitutions were characterised.

**Figure 3:**
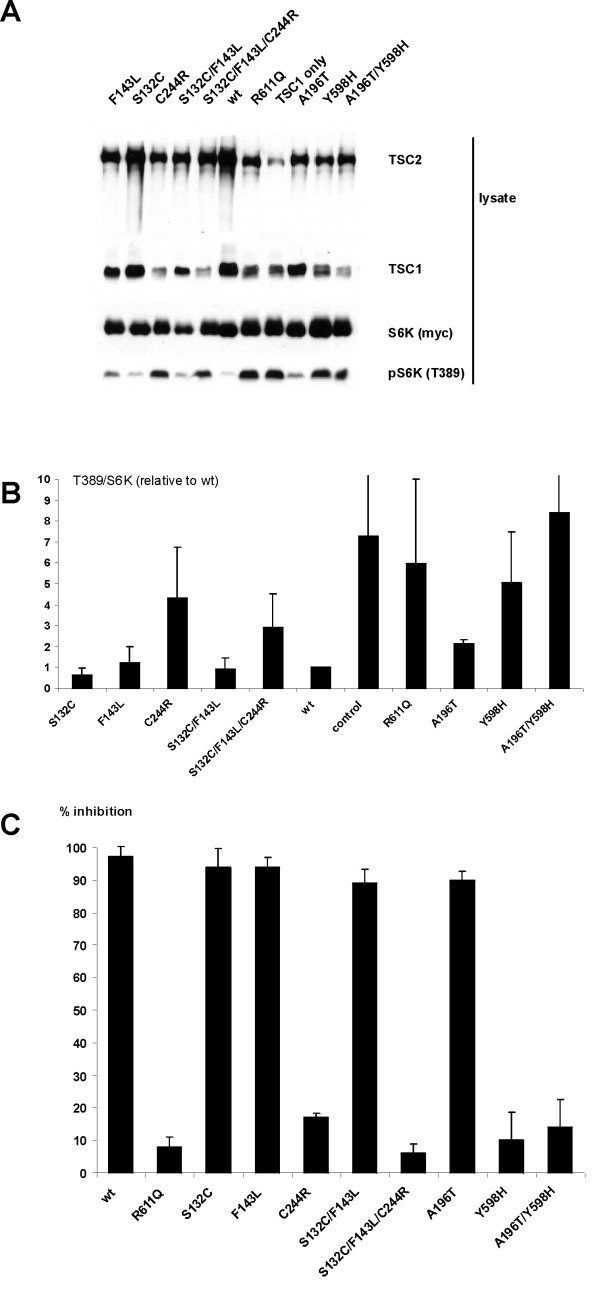
Results of the functional assays on the TSC2 variants identified in family 3 (variants A196T and Y598H) and family 4 (variants S132C, F143L and C244R). (A) TSC2-dependent inhibition of S6K-T389 phosphorylation. S6K, TSC1 and wild-type TSC2 (wt), or S6K, TSC1 and the TSC2 variants, were overexpressed in HEK 293 cells. Phosphorylation of S6K at the T389 position was determined by immunoblotting. A representative example of at least 3 separate experiments is shown. (B) TSC2-dependent inhibition of S6K-T389 phosphorylation. Ratio of total S6K:T389-phosphorylated S6K, as detected by immunoblotting, was estimated by densitometry scanning (Total Scan). Mean ratios relative to TSC2 wild-type (wt) are shown.(C) Inhibition of S6 phosphorylation in *Tsc2 *-/- MEFs. Percentage of *Tsc2 *-/- MEFs transfected with expression constructs encoding TSC1 and wild-type TSC2, or TSC1 and the TSC2 variants, and showing inhibition of S6 phosphorylation. Data from at least 3 separate experiments are shown.

As shown in Figure 3A and 3B, S6K T389 phosphorylation was inhibited by expression of the TSC2 S132C, F143L and A196T single variants, and by the S132C/F143L double variant. In contrast, expression of the C244R and Y598H single variants did not inhibit S6K phosphorylation, and expression of TSC1 was reduced in the presence of these variants. Consistent with these observations, the S132C/F143L/C244R triple variant and A196T/Y598H double variant were unable to inhibit S6K phosphorylation and TSC1 expression was reduced in the presence of these variants, compared to in the presence of wild-type TSC2. To provide confirmation for these data, double-label immunofluorescent microscopy was performed to determine whether S6 phosphorylation was down-regulated in *Tsc2 *-/- MEFs expressing the different variants. As shown in Figure 3C, expression of the TSC2 C244R, S132C/F143L/C244R, Y598H and A196T/Y598H variants was unable to inhibit S6 phosphorylation in the *Tsc2 *-/- MEFs. Therefore, in both assays, the Y598H and C244R variants correspond to the pathogenic mutations in families 3 and 4 respectively.

## Discussion

Mutation analysis of the *TSC1 *and *TSC2 *genes in individuals with TSC, and in those suspected of having the disease, facilitates the diagnosis of TSC, and can help genetic counselling. In most cases, analysis of the patient's DNA results in the identification of a pathogenic mutation. However, in some cases it is impossible to determine from the genetic data alone whether specific, identified changes are pathogenic or not. This can be a particular problem when a change co-segregates with signs of TSC in a single family. To address such cases, we have performed functional analysis of the predicted protein products of the *TSC2 *variants identified in 4 families with TSC.

In families 1 and 2 the index case did not fulfil the diagnostic criteria for TSC and had inherited 2 non-truncating *TSC2 *nucleotide changes, one from each parent. It was not possible to determine from the clinical and genetic data alone whether the families were affected by TSC and, if that was the case, which nucleotide change was the disease-causing mutation.

In families 3 and 4, in which the index case did fulfil the diagnostic criteria for TSC, it was impossible to determine whether the identified TSC2 variant was pathogenic because essential genetic and/or clinical data was not available. In all the above cases we analysed the effect of the identified changes on the activity of the TSC1–TSC2 complex in order to establish which of the changes were pathogenic mutations.

### Families 1 and 2

The *in vitro *GTPase activity of rheb was not stimulated in the presence of the TSC2 I820del and L1511H variants, and neither variant was able to inhibit S6K and S6 phosphorylation as effectively as wild-type TSC2, or the R1772C and T993M variants. In addition, the I820del variant formed a complex with TSC1 less efficiently than wild-type TSC2. The I820del and L1511H changes therefore had similar effects to other pathogenic TSC2 missense mutations, disrupting the function of the TSC1–TSC2 complex *in vitro*. We classified the I820del and L1511H variants as pathogenic mutants. The R1772C and T993M substitutions had no effect on TSC1–TSC2 activity in the assays used and were therefore classified as rare polymorphisms.

*In utero *cardiac rhabdomyoma was the only sign of TSC in the index patient of family 1 (individual II:2). Rhabdomyoma is the most common foetal and neonatal cardiac tumour and although it can be associated with several different genetic disorders, TSC is implicated in as many as two-thirds of the cases [26]. Confirmation of the inactivating nature of the TSC2 I820del mutation identified in family 1 meant that a diagnosis of TSC could be assigned to individuals I:1 and II:2 with more certainty. Previously, the very mild presentation of the disease in individal I:1 had made diagnosis difficult. Mutation analysis, complemented by functional assays, was required to establish whether this individual carried a pathogenic *TSC2 *mutation, and the identification of a mutation in this individual has implications for the family in regard to decisions about having additional children and to the risk of other relatives of the parent. Somatic mosaicism in individual I:1 could not be rigorously excluded, since only leukocyte DNA was available for analysis. Therefore, the recurrence risk for this couple is up to 50%.

Analysis of the TSC2 I820del mutant indicated that the isoleucine residue helps to stabilise TSC1–TSC2 binding. However, since some TSC1–TSC2 binding was detected, and some rheb GAP activity could also be measured, it is possible that the TSC2 I820del variant retains some activity *in vivo*. This provides a possible explanation for the mild symptoms in individual I:1. The second variant identified in family 1, TSC2 R1772C, did not have a deleterious effect on the activity of the TSC1–TSC2 complex. This was somewhat surprising as the R1772 residue is part of the consensus extracellular signal-regulated kinase (ERK) phosphorylation site that has been shown to be involved in regulating the activity of the complex [27,28]. Indeed, SCANSITE [23] indicated that the R1772C substitution destroys the putative ERK phosphorylation site. Furthermore, both the BLOSUM 62 and PAM 250 matrices indicated that the R1772C substitution is likely to have a deleterious effect on TSC2 structure. One possibility is that under certain conditions, the R1772C substitution may have a modifying effect on the TSC1–TSC2 complex, enhancing TSC1–TSC2 activity by preventing the Erk-dependent down-regulation of the complex [28]. This may explain the increased rheb GTPase activity measured in the presence of the TSC2 R1772C variant, compared to wild-type TSC2 (wild-type TSC2 GDP/GTP ratio = 1.4; R1772C GDP/GTP ratio = 1.9; Figure 2B).

Both the 2476delATC (I820del) and 5332C>T (R1772C) variants have been identified in TSC patients by other researchers [29-31]. Strizheva *et al*. [30] suggested that the R1772C substitution could be a pathogenic change while Langkau *et al*. [31] concluded that it was a polymorphism. The status of the I820del change was not certain [29]. Our analysis is consistent with the findings of Langkau *et al*. and, in addition, indicates that the TSC2 I820del variant is pathogenic. Furthermore, this example demonstrates that predictions about the pathogenicity of missense changes based on the results of the BLOSUM 62, PAM 250 and Grantham matrices are not always reliable.

In family 2, mutation analysis resulted in the identification of 2 novel *TSC2 *missense changes, 2996C>T (T993M) and 4550T>A (L1511H), in the index case (individual III:4). Individual III:1, the half-sibling of the index case was later diagnosed with TSC on the basis of the accepted clinical criteria and was found to carry the *TSC2 *4550T>A (L1511H) substitution only. No other nucleotide changes were identified in the *TSC1 *or *TSC2 *genes in either individual. The identification of the TSC2 L1511H change in individual III:1 excluded the T993M change as the common cause of TSC in this family. However, it did not confirm the pathogenicity of the TSC2 L1511H change. The presence of multiple dental pits in both individual II:8 and individual II:10 suggested that the signs of TSC in individuals III:1 and III:4 could be caused by two different mutations, inherited from individuals II:8 and II:12 respectively. However, the functional tests confirmed the pathogenic nature of the TSC2 L1511H substitution, indicating that this was the disease-causing mutation in both cases. The substitution of a basic histidine residue for a nonpolar leucine residue at position 1511 did not affect the TSC1–TSC2 interaction, but did abrogate the rheb GAP activity of the TSC1–TSC2 complex. Although L1511 is outside the predicted TSC2 GAP domain [3], it is clearly necessary for activity. The substitution of a polar threonine for nonpolar methione at position 993 had no deleterious effect on the TSC1–TSC2 interaction or on the activity of the complex. The T993M substitution occurs at a possible site of protein kinase B (PKB)-dependent phosphorylation [27], and may therefore inhibit PKB-mediated inactivation of the TSC1–TSC2 complex. This may explain why the TSC2 T993M variant, like the R1772C variant, had a higher *in vitro *rheb GAP activity than wild-type TSC2 (wild-type TSC2 GDP/GTP ratio = 1.4; T993M GDP/GTP ratio = 1.9; Figure 2B). The T993M polymorphism may therefore also act as a positive modifier of TSC1–TSC2 activity.

In family 3, 2 missense changes (*TSC2 *604G>A (A196T) and *TSC2 *1810T>C (Y598H)) were identified in the index case (individual II:1). Since there was no genetic or clinical data on one of the parents, it was not possible to determine whether either of the changes was responsible for TSC in this individual. Both variants had been identified previously in other TSC patients [29], however in these patients it was also not clear whether the changes were pathogenic. Functional analysis indicated that the TSC2 Y598H substitution reduced TSC1–TSC2 binding and the TSC1–TSC2 dependent inhibition of mTOR activity. In contrast, the A196T substitution did not affect TSC2 activity. Therefore, the Y598H substitution was responsible for TSC in individual II:1, as well as in other TSC patients [29], and the A196T substitution is a rare polymorphism.

In family 4, 3 missense changes were identified in the *TSC2 *gene. The 413C>G (S132C) and 447C>G (F143L) substitutions were inherited from a parent with symptoms suggesting TSC. The third substitution, 748T>C (C244R), was either a *de novo *mutation, or was inherited from the other parent, on whom there was no clinical or genetic data. None of these changes have been described previously [29]. One possibility in this family was that the combination of missense changes was responsible for the TSC phenotype. Therefore we tested all the individual TSC2 single amino acid variants (S132C, F143L and C244R) as well as the TSC2 S132C/F143L double variant and TSC2 S132C/F143L/C244R triple amino acid variant. Neither the S132C nor the F143L changes had a significant effect on TSC1–TSC2 function. In some assays the F143L variant appeared less active than wild-type TSC2, but still had the ability to significantly inhibit mTOR activity. In contrast, the C244R amino acid substitution reduced TSC1–TSC2 binding and was unable to inhibit mTOR activity, either alone or in combination with the S132C and F143L variants. We concluded that the C244R substitution was the pathogenic mutation in the index case. Individual I:1 tested negative for the *TSC2 *748T>C (C244R) substitution, but was diagnosed with angiomyolipomas. Although insufficient for a definite diagnosis of TSC, the incidence of angiomyolipomas is high in the TSC patient population. One possibility is that this individual is a mosaic for the *TSC2 *748T>C (C244R) susbstitution, and that the angiomyolipoma originates from these mosaic cells. An alternative explanation is that the *TSC2 *447C>G (F143L) substitution does have an effect on TSC2 function, sufficient to allow the formation of angiolipoma. The F143L amino acid substitution may therefore be a negative modifier of TSC1–TSC2 activity, in contrast to the R1772C and T993M substitutions identified in families 1 and 2, that, in some assays, appeared to increase TSC1–TSC2 activity. Although there was no evidence in family 1 or family 2 for the non-pathogenic TSC2 variant acting in trans to neutralise the pathogenic variant, it will be interesting to determine whether there are 'hyperactive' TSC1 and TSC2 variants that can (partially) compensate for the presence of pathogenic TSC1 and TSC2 variants. Improving the sensitivity and reliability of the assays of TSC1–TSC2 activity will allow us to more accurately define the activity of different TSC1 and TSC2 variants.

We did not investigate the other putative functions of TSC2 since, in each family, we were able to differentiate between the TSC2 variants on the basis of their inhibition of mTOR signalling. However, it may also be informative to investigate the effects of pathogenic and non-pathogenic amino acid substitutions on the other proposed functions of TSC2, such as the regulation of p27 [32].

Care must always be taken in the interpretation of nucleotide or amino acid changes identified during molecular genetic investigations. As the examples described here demonstrate, both the affection status of individuals in a family and the nature of the nucleotide changes identified in those individuals are not always clear. To distinguish between pathogenic mutations and harmless polymorphisms, we have analysed the effects of different amino acid changes on the activity of the TSC1–TSC2 complex. In each case we have been able to distinguish pathogenic TSC2 variants from rare polymorphic variants, and thereby identify the mutation in each family. We have shown that functional assays can be a useful tool to complement traditional DNA-based mutation analysis in TSC. Identification of the pathogenic mutations in the TSC families described here enabled not only genetic counselling and prenatal testing for future pregnancies but also improved the diagnosis of affected family members to facilitate their critical clinical care. The use of functional assays to differentiate between polymorphisms and pathogenic mutations, in TSC and other diseases, will facilitate not only the identification of pathogenic mutations but will also help establish how different amino acid residues contribute to protein function.

## Conclusion

Deletion of isoleucine at amino acid residue 820 of TSC2 and the TSC2 L1511H, C244R and Y598H amino acid substitutions are sufficient to cause TSC. The TSC2 R1772C, T993M, S132C, F143L and A196T substitutions are rare polymorphisms that do not inhibit TSC1–TSC2 function, and do not cause TSC.

## Abbreviations

DNA, deoxyribonucleic acid; ERK, extracellular regulated kinase; GAP, GTPase activating protein; GDP, guanosine diphosphate; GTP, guanosine triphosphate; HEK 293, human embryonal kidney cells; MEFs, mouse embryo fibroblasts; mTOR, mammalian target of rapamycin; PKB, protein kinase B; rheb, ras homolog expressed in brain; RNA, ribonucleic acid; S6K, p70 S6 kinase; TSC, tuberous sclerosis complex.

## Competing interests

The author(s) declare that they have no competing interests.

## Authors' contributions

MN, OS, MG and AA performed the practical work; MW, AM-K, MB and CD co-ordinated the clinical investigations of the patients; AvdO and DH led the research. The manuscript was drafted by OS, MN, AvdO and DH, and read and approved by all authors.

## Pre-publication history

The pre-publication history for this paper can be accessed here:


